# Application of genomic selection and experimental techniques to predict cell death and immunotherapeutic efficacy of ferroptosis-related CXCL2 in hepatocellular carcinoma

**DOI:** 10.3389/fonc.2022.998736

**Published:** 2022-10-05

**Authors:** Qiaoli Yi, Qiuju Liang, Yuanhong Liu, Zhicheng Gong, Yuanliang Yan

**Affiliations:** ^1^ Department of Pharmacy, Xiangya Hospital, Central South University, Changsha, China; ^2^ National Clinical Research Center for Geriatric Disorders, Xiangya Hospital, Central South University, Changsha, China

**Keywords:** ferroptosis, hepatocellular carcinoma, CXCL2, immune infiltration, prognosis

## Abstract

Since most hepatocellular carcinoma (HCC) patients are diagnosed at advanced stages, there is no effective treatment to improve patient survival. Ferroptosis, a regulated cell death driven by iron accumulation and lipid peroxidation, has been reported to play an important role in tumorigenesis. However, the detailed mechanism and biological function of ferroptosis are still incompletely understood in HCC patients. In this study, we analyzed genomic profiles of three HCC datasets, GSE6764, GSE14520, and GSE14323. Venn diagrams were implemented to visualize the overlapping genes between differentially expressed genes and ferroptosis-related gene set. Then, one up-regulated gene, ACSL4, and five down-regulated genes, STEAP3, MT1G, GCH1, HAMP, and CXCL2, were screened. Based on the survival analysis performed by Kaplan-Meier plotter database, ferroptosis-related gene CXCL2 was demonstrated positively-correlated with the patients’ prognosis. Moreover, CXCL2 overexpression significantly inhibited cell growth and improved cellular ROS, Fe^2+^ and MDA levels in HCC cells Huh7 and MHCC97H, suggesting the roles of CXCL2 in inducing ferroptotic cell death. In addition, aberrantly expressed CXCL2 was negatively associated with malignancy clinical features, such as nodal metastasis and higher grades. The ssGSEA enrichment analysis revealed that CXCL2 co-expressed molecules were mainly involved in inflammation and immune-related pathways, such as acute inflammatory response, humoral immune response, adaptive immune response. TISIDB algorithm indicated the positive correlation between CXCL2 expression and tumor-infiltrating immune cells, including neutrophils and macrophages. Additionally, we also found that CXCL2 was positively correlated with immune infiltration score, and HCC patients with higher score harbored better prognosis. Together, these findings suggested that CXCL2 may enhance ferroptosis sensitivity and regulate immune microenvironment in HCC, and serve as a promising prognosis biomarker for HCC patients.

## Introduction

Liver cancer is the third leading cause of cancer-related mortality worldwide after lung cancer and colorectal cancer, and hepatocellular carcinoma (HCC) accounts for approximately 90% of all cases (1, 2). The main risk factors for HCC include chronic viral infection by hepatitis B virus (HBV) or hepatitis C virus (HCV), habitual alcohol consumption, and non-alcoholic steatohepatitis (NASH) associated with metabolic syndrome, indicating that inflammation has an important function in the development of HCC (3, 4). Patients with HCC are often diagnosed at advanced stages, which contributes to its poor prognosis. Currently, surgical resection, liver transplantation, trans-arterial chemoembolization, local ablation, and systemic therapy are the major therapeutic modalities for HCC (5). Immunotherapy is emerging as a promising and effective therapeutic approach among systemic therapy after sorafenib. Nivolumab is the first anti-programmed death-1 (PD-1) antibody approved by the United States Food and Drug Administration (FDA) as a second-line treatment for patients with advanced HCC (6). Furthermore, clinical trials evaluating PD-1 blockade as first-line treatment strategy in HCC are underway (NCT02576509). However, it is only a small subset of patients treated with immune checkpoint inhibitors that benefit from these agents (7). Therefore, it is urgently necessary to identify novel potential biomarkers to improve patient response rates and survival.

Ferroptosis is a novel form of regulated cell death driven by iron-dependent lipid peroxidation (8, 9). Accumulating evidence suggests that ferroptosis has significant implications on tumorigenesis and cancer progression (10, 11). P53, one of the most extensively studied tumor suppressor genes, promotes ferroptosis pathway by repressing the expression of solute carrier family 7 member 11 (SLC7A11), a pivotal component of the cystine/glutamate antiporter. Moreover, SLC7A11 is up-regulated in human tumors, and its upregulation inhibits reactive oxygen species (ROS)–induced ferroptosis and abrogates p53^3KR^ (an acetylation-defective mutant)-mediated tumor growth suppression (12, 13). Ferroptosis induction in combination of other cancer treatments might enhance the therapeutic response in patients by increasing drug sensitivity (14, 15). However, the possible roles and underlying mechanisms of ferroptosis in HCC remain incompletely characterized.

C-X-C motif chemokine ligand 2 (CXCL2) is a member of chemokine superfamily, which encodes secreted proteins participated in inflammatory processes and immunoregulatory (16, 17). Moreover, increasing evidence indicates that CXCL2 is also involved in tumor initiation and progression. A recent study reported that elevated CXCL2 in the tumor microenvironment promoted the recruitment of myeloid-derived suppressor cells and was correlated with poor prognosis in patients with bladder cancer (18). Intriguingly, Ding and the colleague revealed that CXCL2 expression was down-regulated in HCC and overexpression of CXCL2 inhibited tumor cell proliferation and promoted apoptosis (19). However, the underlying mechanisms of CXCL2 in inflammation and HCC progression remains to be further investigated.

In this study, we comprehensively analyzed the biological functions of CXCL2 in HCC. According to several genomic selection strategies, CXCL2, a ferroptosis-related gene, was found to be down-regulated in HCC and influence tumor progression and clinical prognosis of HCC patients. Furthermore, we explored the possible roles of CXCL2 in inducing ferroptotic cell death. These results indicated that CXCL2 harbored vast potential significance as a prognostic biomarker and therapeutic target for patients with HCC.

## Materials and methods

### Multi-omics data collection

Three HCC datasets, GSE6764 (20), GSE14520 (21), and GSE14323 (22), were screened according to the inclusion criteria detailed in a previous study by our research group (23). Detailed characteristics of the three GEO datasets were shown in **Table 1**. Differentially expressed genes (DEGs) between HCC tumor samples and normal tissues were identified based on the criteria: P < 0.01 and |Log2 FC (Fold Change)| > 1. Moreover, 259 ferroptosis-related genes were downloaded from FerrDb (http://www.zhounan.org/ferrdb/legacy/index.html) (24). Next, Venn diagrams (http://bioinformatics.psb.ugent.be/webtools/Venn/) were generated to identify the co-DEGs among three GEO datasets and ferroptosis-related gene dataset.

**Table 1:** Detailed characteristics of the three GEO datasets in our study.

GEO datasets	Platform	Sample type	Sample size (tumor/control)	References
GSE6764	GPL570	tissue	75 (35/40)	(20)
GSE14520	GPL571 GPL3921	tissue	43 (22/21) 445 (225/220)	(21)
GSE14323	GPL96 GPL571	tissue	9 (9/0) 115 (55/60)	(22)

### Genomic selection analyses

The integrative bioinformatics analysis was performed using several online bioinformatics databases (**Table 2**).

**Table 2:** The bioinformatics databases analyzed in this study.

Database	URL	References
Kaplan-Meier Plotter	https://kmplot.com/analysis/	(25)
GEPIA2	http://gepia2.cancer-pku.cn/#index	(26)
TNMplot	https://tnmplot.com/	(27)
UALCAN	http://ualcan.path.uab.edu/	(28)
LinkedOmics	http://linkedomics.org/login.php	(29)
TISIDB	http://cis.hku.hk/TISIDB	(30)
TIDE	http://tide.dfci.harvard.edu/	(31, 32)

We employed the Kaplan-Meier plotter (25) and GEPIA2 database (26) to assess the prognostic values of co-DEGs in HCC patients, including overall survival (OS), progression-free survival (PFS) and disease-specific survival (DSS). The expression levels of CXCL2 in GSE6764, GSE14520, and GSE14323 were analyzed by using the GEO2R algorithm (https://www.ncbi.nlm.nih.gov/geo/geo2r/). Furthermore, the expression pattern of CXCL2 between HCC tumor samples and normal tissues was cross-validated by the TNMplot (27), Xiantao tool (https://www.xiantao.love/products) and UALCAN (28). Xiantao tool is a comprehensive bioinformatics toolbox to perform differential expression analysis, functional enrichment analysis, interaction networks, and clinical prognosis across different cancer types from The Cancer Genome Atlas (TCGA) database. We used Xiantao toolbox and UALCAN to assess the association between CXCL2 and clinical pathological parameters in TCGA-LIHC cohort.

LinkedOmics could be used to analyze the multi-omics data across various cancer types, with three analytical algorithms: LinkFinder, LinkInterpreter, and LinkCompare (29). The heatmaps of the top 50 genes positively and negatively correlated with CXCL2 were analyzed with the LinkFinder module. Furthermore, Gene Ontology (GO) and Kyoto Encyclopedia of Genes and Genomes (KEGG) pathway analysis were implemented using the LinkInterpreter algorithm.

Next, we performed the single sample Gene set enrichment analysis (ssGSEA) (33) to assess the correlation between CXCL2 expression and 24 immune cell types in TCGA-LIHC. In addition, we used the TISIDB (30) to validate the roles of CXCL2 in immune-related responses, such as tumor-infiltrating immune cells, and immunomodulators. In addition, Tumor Immune Dysfunction and Exclusion (TIDE) (31, 32) was applied to predict the roles of CXCL2 in immunotherapy response of HCC patients.

### Cell cultures and reagents

Human HCC cells, Huh7 and MHCC97H, and human immortalized hepatocyte, HHL-5, were kindly provided from the Cancer Research Institute of the Central South University (Changsha, China) and cultured in DMEM (C11995500, HyClone, USA) supplemented with 10% fetal bovine serum (04-001-1A, BI, Israel) and 1% penicillin and streptomycin (10378016, Gibco, USA) at 37°C with 5% CO2. The overexpression plasmid HY21177 pcDNA3.1-CXCL2 (NM_002089)-3xFlag-C plasmid was purchased from Guangzhou Dahong Biological Technology Co., Ltd. (China). The CXCL2 overexpression plasmid was extracted with a SanPrep Column Plasmid Mini-Preps kit (Sangon Biotech, Shanghai, China), and then transfected into Huh7 and MHCC97H cell lines for 24 h using Lipofectamine 3000 (L300015, Thermo Fisher Scientific, USA) following the manufacturer’s instructions.

### RNA isolation and real-time PCR

Total RNA was extracted from cells with TRIzol reagent (Invitrogen, USA), and then reverse-transcribed into cDNA using a PrimeScriptTM RT reagent kit (RR047A, Takara, China) with gDNA Eraser (Perfect Real Time) according to the manufacturer’s protocol. The qPCR reaction was performed with iTaqTM Universal SYBR green Supermix (1725121, Bio-Rad, USA). Relative RNA levels were calculated using the 2-ΔΔct method with RNA levels of GAPDH used as internal controls. The sequences of gene-specific primers are listed as follows: CXCL2 forward: 5’-GCTTGTC TCAACCCCGCATC-3’ and reverse: 5’-TGGATTTGCCATTTTTCAGCATCTT-3’; GAPDH forward: 5’-ACAGCCTCAAGATCATCAGC-3’ and reverse: 5’-GGTCATGAGTCCTTCCACGAT-3’.

### Western Blot

The cultured cell lines were collected and then lysed with RIPA lysis buffer (20101ES60, Yeasen Biotech, China) supplemented with proteinase inhibitors (B14012, Bimake, USA). Equal amounts of total protein (50 µg) were loaded into each lane of 15% SDS–polyacrylamide gel electrophoresis (PAGE). Subsequently, proteins were transferred to PVDF membranes (0.22 µm: ISEQ00010; 0.45 µm: IPVH00010). Next, membranes were blocked in 5% skimmed milk at RT for 1 h, and then incubated overnight at 4 °C with primary antibodies in 5% Bovine Serum Albumin (D620272, Sangon Biotech, China), followed by HRP-conjugated secondary antibody (1:3000; SA00001-2, proteintech) at RT for 1 h. Primary antibodies are described as follows: CXCL2 (1:1000; bs-1162R, Bioss); Actin (1:2000; sc-58673, Santa Cruz Biotechnology). Finally, proteins were visualized using Immobilon Western Chemiluminescent HRP Substrate (WBKLS0500, Millipore, USA).

### Tissue microarray and immunohistochemistry

Tissue microarrays (TMAs) containing 80 pairs of HCC and matched paracancerous tissues were purchased from shanghai Outdo biotechnology company Ltd. (HLivH160CS02, Shanghai, China). Immunohistochemistry (IHC) staining of CXCL2 was conducted using a Histomouse SP Kit (959551, Invitrogen, USA) according to the manufacturer’s protocal. The concentration of antibody against CXCL2 was 1:100. The results of CXCL2 staining in tissues were independently evaluated by two pathologists. The evaluation of proportion score was on a scale of 1-4 (1, 0%-25%; 2, 25.1%-50%; 3, 50.1%-75%; 4, 75.1%-100%). The staining intensity score was graded as follows: 0, negative; 1, weak; 2, moderate; 3, strong. Then the histologic score for each tissue was calculated with the formula: histologic score = proportion score × intensity score.

### Iron assay

The concentration of ferrous iron (Fe^2+^) was measured using an iron colorimetric assay kit (ab83366, Abcam, USA) according to the manufacturer’s instructions. After CXCL2 or control plasmids overexpression, Huh7 and MHCC97H cells were treated with erastin (10 μM) for 24 hours. Cells were harvested using trypsin without EDTA and homogenized in iron assay buffer on ice, then centrifuged at 4°C (14,000×g, 15 min) to remove insoluble material. Subsequently, collect the supernatant and add assay buffer, mix and incubate for 30 min at 25°C. Add 100ul iron probe into each sample and incubate at 25°C for 60 min protected from light. Detect the absorbance at 593 nm using the VICTOR X2 microplate reader (PerkinElmer, Waltham, USA).

### Malondialdehyde assay

The relative MDA concentration was determined using a lipid peroxidation assay (MAK085, Sigma, USA) according to the manufacturer’s protocol. Cells were processed with CXCL2 overexpression plasmids or erastin as described previously, then collected and homogenized in MDA lysis buffer with BHT on ice. Centrifuge the samples at 13,000 × g for 10 minutes to remove insoluble material. Collect the supernatant and add thiobarbituric acid (TBA) into each sample. Then incubate the samples at 95 °C for 60 min to form the MDA-TBA adduct. Measure the absorbance at 532 nm using the VICTOR X2 microplate reader (PerkinElmer, Waltham, USA).

### ROS assay

Intracellular ROS level was evaluated by CytoFLEX flow cytometry (Beckman Coulter, USA). Briefly, about 10^5^ cells were collected after CXCL2 overexpression plasmids or erastin treatment as described previously, and then stained with the oxidation-sensitive fluorescent probe dye 2′,7′-dichlorodihydrofluorescein diacetate (DCFDA; Abcam, ab113851) according to the manufacturer’s instructions. Finally, flow cytometry analysis was performed using FlowJo software (v10.8.1, USA).

### Cell counting kit 8

Huh7 and MHCC97H cells were transfected with pcDNA3.1-CXCL2 overexpression plasmid or empty pcDNA3.1 (+) plasmid as control for 24 h and then seeded in 96-well culture plates (2 × 10^3^ cells/well). At 24h, 48h, 72h, 96h, and 120h, cell viability was assessed by performing CCK-8 assay (B34304, Bimake, USA) reading absorbance at 450 nm using a VICTOR X2 microplate reader (PerkinElmer, USA) according to the manufacturer’s protocols.

### Colony formation assay

Huh7 and MHCC97H cells were transfected as previously described and then seeded in 6-well plates (10^3^ cells/well). After incubating at 37°C for about 14 days, the cells were washed twice with PBS and then stained with 0.3% w/v crystal violet/methanol for 15-20 min at room temperature (RT).

### Statistical analysis

All experiments and assays were independently repeated by at least three times and results were reported as means ± standard deviations (SD). Statistically significant differences were performed using Student’s t-test or ANOVA. Kaplan–Meier survival analysis was assessed by log-rank test. The immune score, stromal score, and ESTIMATE score of each tumor sample were estimated using the R package “ESTIMATE” based on expression data (34). Statistical analysis was carried out using GraphPad Prism 8 and P < 0.05 was considered as statistically significant difference.

## RESULTS

### Identification of differentially expressed genes

We analyzed the gene expression profiles of three HCC datasets (GSE6764, GSE14323, and GSE14520) and screened the DEGs between HCC and normal liver tissues according to the screening criteria: P < 0.01 and | log2 FC| > 1. We identified 826 up-regulated genes in GSE6764, 332 in GSE14323, and 505 in GSE14520, respectively. Meanwhile, 859 genes in GSE6764, 257 in GSE14323, and 583 in GSE14520 had been identified to be significantly down-regulated in HCC (**Supplementary Table S1**). And 259 ferroptosis-related genes were downloaded from the FerrDb database.

**Figure 1:**
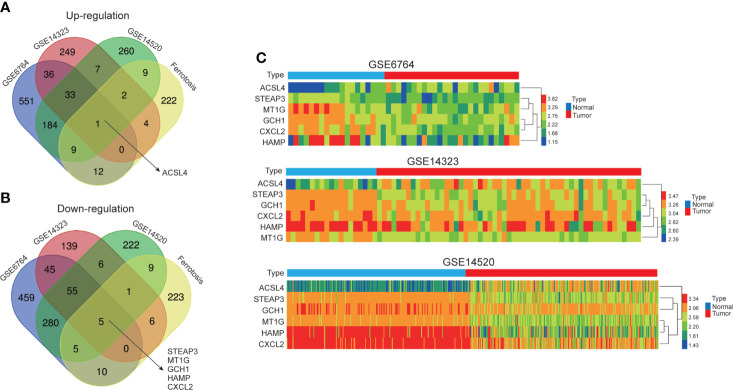
Identification of co-differentially expressed genes (co-DEG). **(A, B)** Venn diagrams exhibited one up-regulated gene, ACSL4, and five down-regulated genes, STEAP3, MT1G, GCH1, HAMP, and CXCL2, between three HCC GEO datasets and ferroptosis-related gene set. The number in overlapping area represents the number of genes. **(C)** The gene expression heatmaps of co-DEGs in GSE6764, GSE14323, GSE14520.

Recently, increasing evidence suggests that ferroptosis has significant implications on tumorigenesis and cancer progression, and ferroptosis induction might ameliorate antitumor efficacy by increasing drug sensitivity (35, 36). In order to explore the roles of ferroptosis in HCC, we employed Venn diagrams to identify co-DEGs between three GEO datasets and ferroptosis-related gene set. As shown in **Figures 1A, B**, one up-regulated gene, ACSL4, and five down-regulated genes, STEAP3, MT1G, GCH1, HAMP, and CXCL2, were preliminarily screened, and the gene expression heatmaps of these co-DEGs in each GEO dataset were presented in **Figure 1C**. These six selected genes were presumed to have potential roles in the occurrence and development of HCC.

### CXCL2 shows the promising prognostic value in HCC

**Figure 2:**
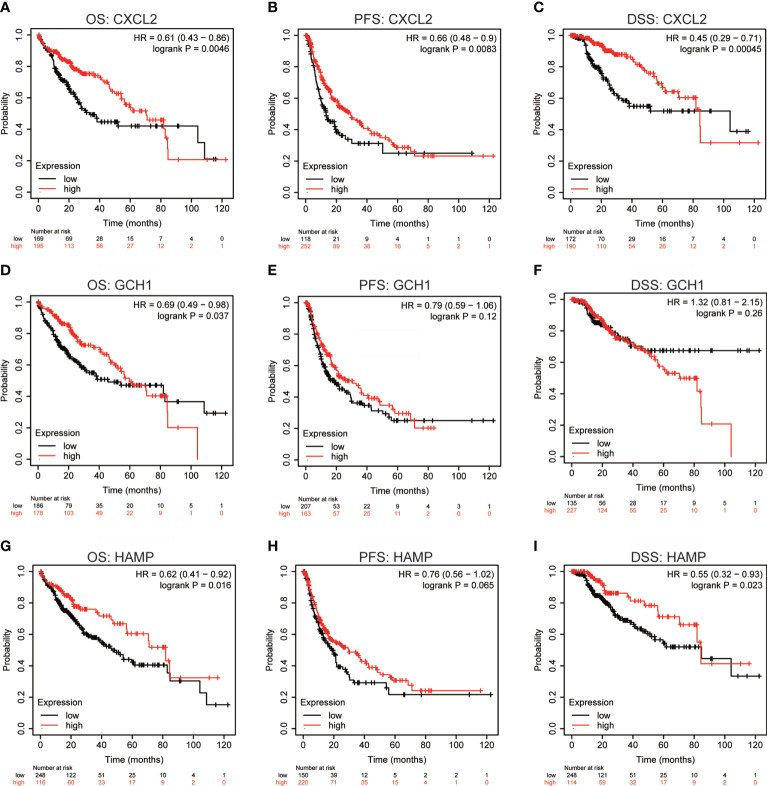
Prognostic values of CXCL2, GCH1, and HAMP in HCC. **(A-I)** Overall survival (OS), progression-free survival (PFS), and disease-specific survival (DSS) Kaplan-Meier curves of CXCL2, GCH1, and HAMP in patients with HCC by Kaplan–Meier plotter database.

Using 60 ferroptosis-related genes dataset, previous study analyzed the diagnostic and prognostic roles of STEAP3, ACSL4 and MT1G in HCC (23). In this study, the correlations between the expression levels of GCH1 (RNAseq ID: 2643), HAMP (RNAseq ID: 57817), and CXCL2 (RNAseq ID: 2920) and prognosis in HCC patients were analyzed using the Kaplan–Meier plotter database. The expression of CXCL2 was significantly associated with favorable OS (HR = 0.61, 95% CI = 0.43–0.86, P = 0.0046), PFS (HR = 0.66, 95% CI = 0.48–0.90, P = 0.0083), and DSS (HR = 0.45, 95% CI = 0.29–0.71, P = 0.00045), which was consistent with low expression of CXCL2 in HCC tissues. However, there was no obvious relationship between the expression of GCH1 or HAMP and prognosis in HCC patients (P > 0.05) (**Figures 2A–I**). In addition, we employed the GEPIA2 database to cross-validate the prognostic value of CXCL2, GCH1, and HAMP, and drew consistent conclusions (**Supplementary Figure S1**). Therefore, these results revealed that CXCL2 expression might associated with clinical outcomes in HCC and warrants further investigation.

### Low expression of CXCL2 in HCC and its correlation with clinicopathologic characteristics

**Figure 3:**
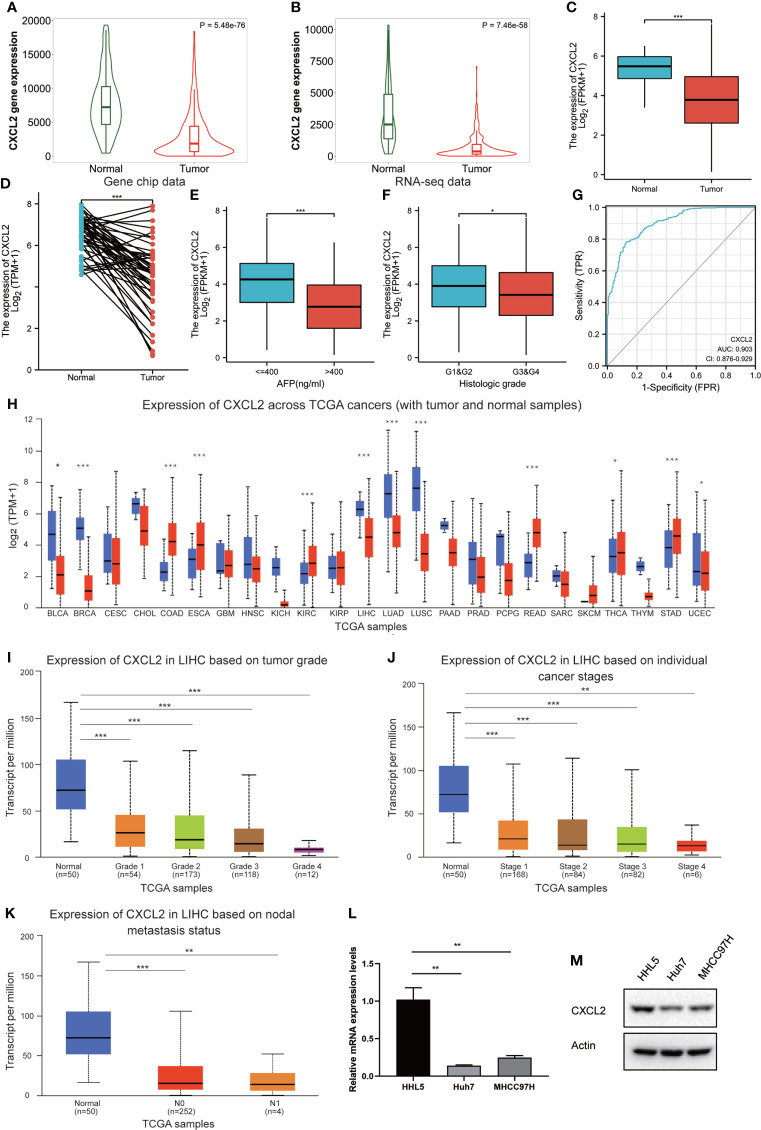
The expression level and clinicopathologic characteristics of CXCL2 in HCC. **(A, B)** Low expression of CXCL2 in HCC samples in gene chip data and RNA-seq data of TNMplot database. **(C, D)** The validation of low expression of CXCL2 in HCC samples in TCGA-LIHC cohort **(C)** or in comparison with matched adjacent para-tumor tissues **(D)** by Xiantao tool. **(E, F)** Correlation of CXCL2 expression with AFP **(E)** and histologic grade **(F)**. **(G)** Receiver operation characteristic (ROC) curve to evaluate the diagnostic value of CXCL2 in HCC. **(H)** Expression pattern of CXCL2 across diverse TCGA cancer types by UALCAN database. **(I-K)** Correlation of CXCL2 expression with tumor grade **(I)**, stage **(J)**, and nodal metastasis **(K)**. **(L, M)** Low expression of CXCL2 in two HCC cell lines, Huh7 and MHCC97H, was further confirmed by real-time PCR **(L)** and western blotting **(M)**, compared with the normal hepatocyte cell line HHL-5. * represents P < 0.05, ** represents P < 0.01, *** represents P < 0.001.

The expression profiles of CXCL2 were further confirmed by several independent online bioinformatics databases, such as TNMplot, Xiantao tool, and UALCAN. Firstly, TNMplot revealed that CXCL2 mRNA expression levels were significantly down-regulated in HCC samples from gene chip data and RNA-seq data (**Figures 3A, B**). Next, Xiantao tool also exhibited the low expression of CXCL2 in HCC tissues compared to normal liver tissues including non-cancerous patients (**Figure 3C**) or matched adjacent para-tumor tissues (**Figure 3D**). We then analyzed the correlations between CXCL2 expression level and clinicopathological characteristics in HCC patients. As shown in **Figures 3E, F**, CXCL2 expression levels were significantly correlated with AFP (alpha-fetoprotein) (P < 0.001) and histologic grade (P = 0.048). Other clinicopathological features of CXCL2 expression in HCC were exhibited in **Supplementary Table S2**. In addition, we explored the diagnostic value of CXCL2 in HCC with receiver operation characteristic (ROC) curve, and the area under the ROC curve (AUC) was 0.903 (**Figure 3G**). This result indicated that CXCL2 might be a potential diagnostic biomarker in HCC patients. Then, we further validated the expression of CXCL2 and its correlation with clinicopathologic characteristics with UALCAN database. **Figure 3H** showed the expression pattern of CXCL2 across diverse TCGA cancer types, and low expression of CXCL2 was associated with malignancy clinical features, such as tumor grade, stage, and nodal metastasis (**Figures 3I–K**). Finally, the low expression of CXCL2 in two HCC cell lines, Huh7 and MHCC97H, was further confirmed by real-time PCR and western blotting, compared with the normal hepatocyte cell line HHL-5 (**Figures 3L, M**).

**Figure 4:**
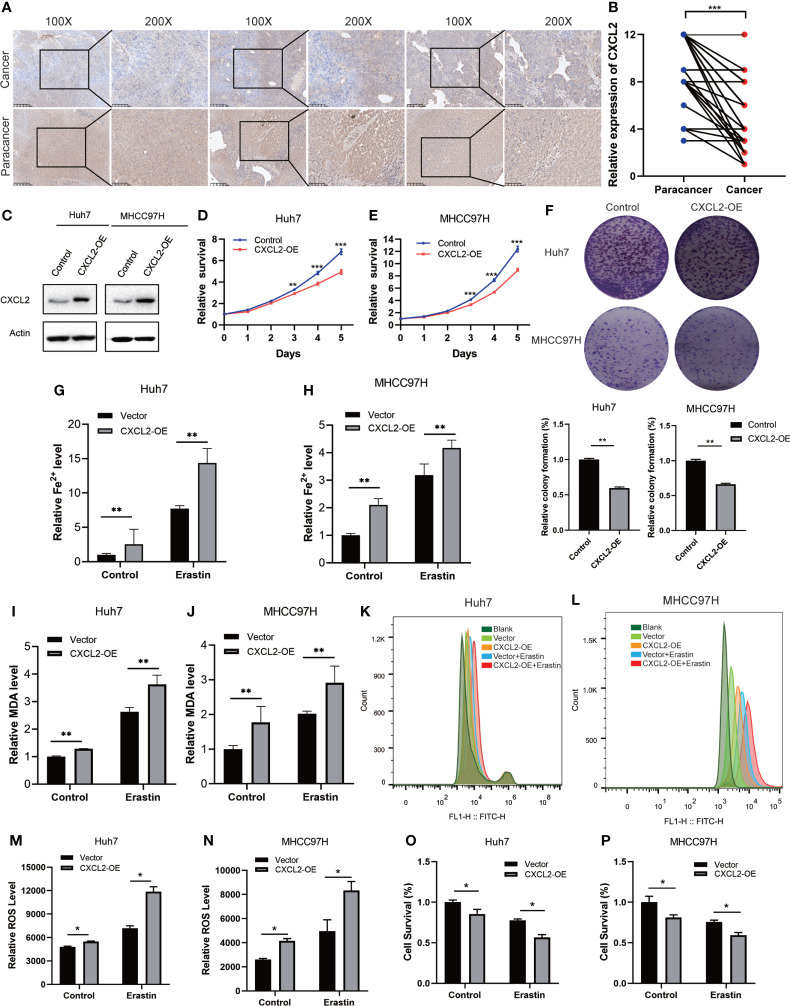
CXCL2 overexpression inhibited cell proliferation. **(A)** IHC images of CXCL2 in paired HCC and paracancerous tissues. **(B)**The histologic score of CXCL2 expression in paired HCC and paracancerous tissues. **(C)** Western blotting validated the overexpression of CXCL2 in two HCC cell lines, Huh7 and MHCC97H. **(D-F)** CCK-8 and colony formation assay revealed that CXCL2 overexpression inhibited cell growth and proliferation. **(G-J)** Fe^2+^ and MDA levels were detected in Huh7 and MHCC97H cells transfected with CXCL2 overexpression plasmids. **(K-N)** Intracellular ROS levels were elevated in Huh7 and MHCC97H cells with CXCL2 overexpression. **(O, P)** CCK-8 assay suggested that the combination of CXCL2 overexpression and erastin significantly inhibited cell survival in Huh7 and MHCC97H cells. * represents P < 0.05, ** represents P < 0.01, *** represents P < 0.001.

The expression of CXCL2 was then examined with IHC staining in tissue microarrays containing 80 pairs of HCC and matched paracancerous tissues, and the results confirmed that HCC tissues harbored significantly lower levels of CXCL2 than paracancer tissues (**Figures 4A, B**). To further explore the role of CXCL2 in HCC, we overexpressed CXCL2 with the overexpression plasmid in Huh7 and MHCC97H cell lines (**Figure 4C**). CCK-8 and colony formation assay indicated that CXCL2 overexpression significantly repressed cell growth and proliferation compared with control group (**Figures 4D–F**). Ferroptosis is a form of regulated cell death characterized by increased intracellular Fe^2+^ and lipid peroxidation, and the marker of lipid peroxidation is MDA (37). After CXCL2 overexpression, the levels of intracellular Fe^2+^ and MDA were significantly increased in Huh7 and MHCC97H cells compared with vectors (**Figures 4G–J**). Similarly, flow cytometry analysis indicated that CXCL2 overexpression elevated intracellular ROS levels with or without erastin (**Figures 4K–N**). CCK-8 assay suggested that the combination of CXCL2 overexpression and erastin significantly inhibited cell survival in Huh7 and MHCC97H cells (**Figures 4O, P**). These findings suggested that CXCL2 overexpression might suppress cell survival in HCC by promoting ferroptosis.

### CXCL2 co-expression network in HCC

**Figure 5:**
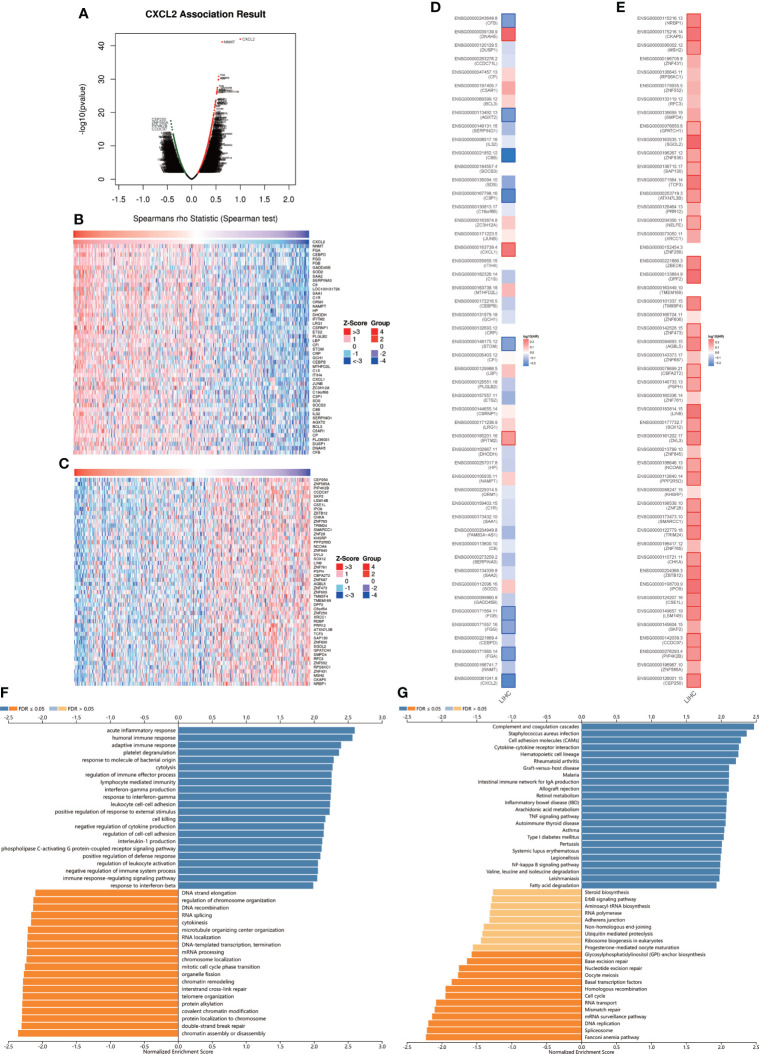
The co-expression network of CXCL2 in HCC. **(A)** Volcano plot for genes positively or negatively associated with CXCL2 in TCGA-LIHC cohort by LinkedOmics database. Red dots represent genes positively correlated with CXCL2, and green dots represent negative correlation **(B, C)** Heatmaps of the top 50 genes positively and negatively correlated with CXCL2 in HCC, respectively. **(D, E)** survival maps of the top 50 genes harboring positive and negative correlations with CXCL2 in HCC. **(F, G)** GO and KEGG pathways of CXCL2-associated network in TCGA-LIHC cohort.

To explore the biological roles of CXCL2 in HCC, we performed the co-expression profile of CXCL2 in the TCGA-LIHC cohort by the LinkFinder module of LinkedOmics. As can be seen from **Figure 5A**, 4643 genes (red dots) were positively related with CXCL2, and 4232 genes (green dots) were negatively associated with CXCL2. **Figures 5B, C** exhibited the heatmaps of the top 50 genes positively and negatively correlated with CXCL2, respectively (**Supplementary Tables S3**, **S4**). Notably, the top 50 positively correlated genes owned a high probability of being low-risk markers in HCC, of which 9/50 genes harbored protective hazard ratio (HR). Contrarily, there were 33 of the top 50 negatively associated genes with unfavorable HR (**Figures 5D, E**).

In addition, we further conducted functional enrichment analysis by the LinkInterpreter module of the LinkedOmics database. GO-biological process showed that genes co-expressed with CXCL2 mainly participated in the inflammation and immune-related terms, such as acute inflammatory response, humoral immune response, adaptive immune response, response to molecule of bacterial origin (**Figure 5F**). KEGG pathway analysis showed that these co-expressed genes were mainly involved in complement and coagulation cascades, staphylococcus aureus infection, cell adhesion molecules, cytokine-cytokine receptor interaction, etc. (**Figure 5G**). In addition, we performed GO and KEGG enrichment analysis in the GSE14520, and the results were similar with the original results (**Supplementary Figure S2**). Taken together, these results suggested that CXCL2-associated network might have a significant impact on inflammation and immune regulation in HCC.

### Role of CXCL2 in the immune microenvironment of HCC

**Figure 6:**
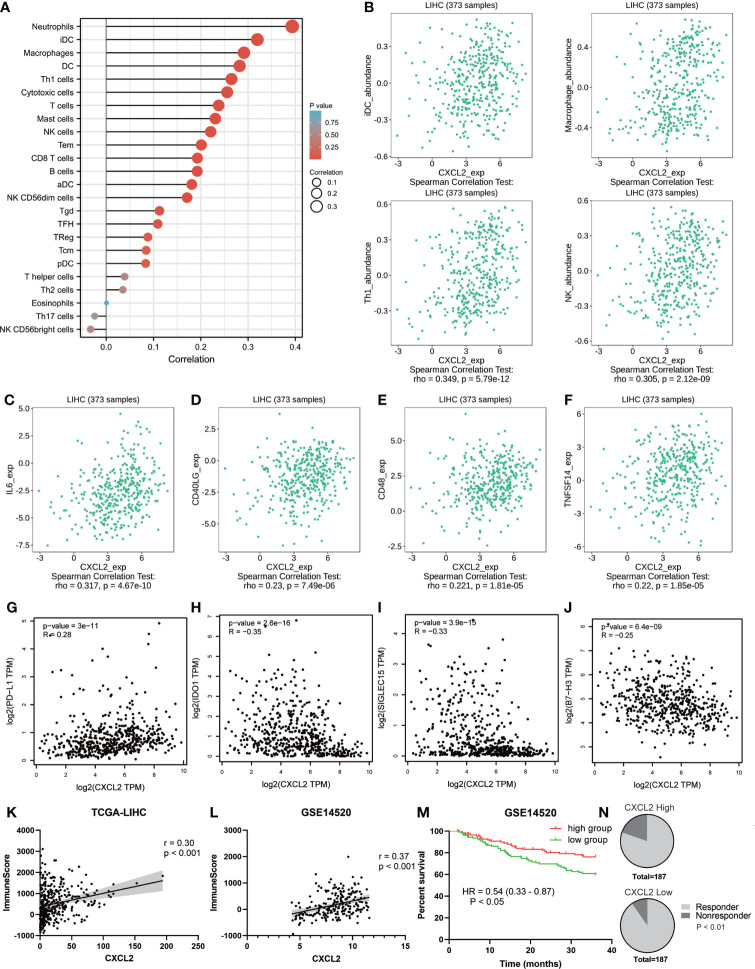
The role of CXCL2 in immune microenvironment of HCC. **(A)** Lollipop diagram exhibiting tumor-infiltrating immune cells associated with CXCL2 by Xiantao tool. **(B)** Scatter plots cross-validating the associations between CXCL2 expression and several TILs, including iDC, macrophage, Th1, and NK cells. **(C-F)** Positive correlations between CXCL2 expression and several immunostimulators, including IL-16, CD40LG, CD48, and TNFSF14. **(G-J)** Association between CXCL2 expression and immune checkpoints. **(K, L)** Association between CXCL2 expression and immune score in TCGA-LIHC cohort and GSE14520 evaluated by ESTIMATE algorithm. **(M)** Kaplan-Meier survival curves showing 3-year overall survival based on immune score in GSE14520 dataset. **(N)** The predicted response rate of the CXCL2 high expression group was lower than that of the CXCL2 low expression group.

Increasing evidence suggests that ferroptosis has great potential in regulating tumor immune microenvironment (38, 39). Hence, we explored the role of ferroptosis-related gene CXCL2 in HCC immune microenvironment through Xiantao tool. As presented in **Figure 6A**, CXCL2 expression was positively associated with the abundance of several tumor-infiltrating immune cells, including neutrophils, immature dendritic cell (iDC), macrophages, type 1 T helper cell (Th1), and natural killer (NK) cells. Similar lymphocyte infiltration profiles were attained by TISIDB database (**Figure 6B**). We continued to analyze the correlation between CXCL2 expression and immunostimulators. **Figures 6C–F** exhibited immunostimulators positively correlated with CXCL2, including interleukin 16 (IL-16), CD40 ligand (CD40LG), CD48, and TNF superfamily member 14 (TNFSF14). Given the clinical implications of checkpoint blockade-based immunotherapy in HCC (3), we further explored the associations between CXCL2 expression and several immune checkpoints. As shown in **Figures 6G–J**, CXCL2 expression was positively associated with programmed cell death ligand 1 (PD-L1), and negatively associated with indoleamine 2,3-dioxygenase 1 (IDO1), sialic acid binding Ig like lectin 15 (SIGLEC15), and B7-H3 (CD276). Additionally, **Figures 6K, L** exhibited positive correlations between CXCL2 and immune infiltration score in TCGA-LIHC cohort and GSE14520 dataset. Patients with high immune infiltration score had better 3-year OS in GSE14520 (**Figure 6M**). In addition, we employed the TIDE algorithm to predict the immunotherapy response of HCC patients based on pre-treatment expression profiles. As shown in **Figure 6N**, the response rate of CXCL2 high expression group predicted by the TIDE database was lower than that of CXCL2 low expression group. This inconsistency with the results of “ESTIMATE” algorithm may be that TIDE database focused on predicting the efficacy of anti-PD1 and anti-CTLA4 therapies. Together, these results suggested that ferroptosis-related gene CXCL2 might affect the prognosis of HCC patients by regulating the immune microenvironment.

### CXCL2 relating M1 Macrophages in HCC

**Figure 7:**
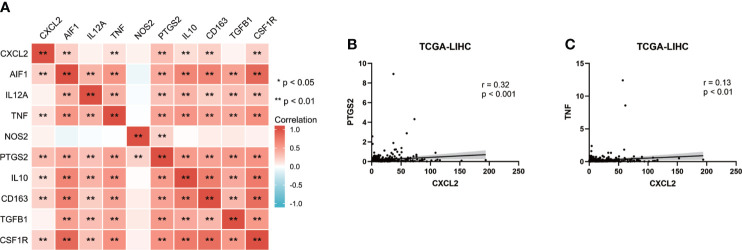
CXCL2 associated with M1 macrophage in HCC. **(A)** Heat map of correlation between CXCL2 and classical macrophage phenotype markers. **(B, C)** Scatter plots showing the association between CXCL2 and M1 macrophage markers (PTGS2 and TNF).

The above results revealed that CXCL2 was positively correlated with macrophage infiltration. We then analyzed the associations between CXCL2 expression and classical macrophage phenotype markers of M0 (undifferentiated) (AIF1), M1 (anti-tumor) (IL12A, TNF, NOS2, PTGS2) and M2 (tumor-promoting) (IL10, CD163, TGFB1, CSF1R) in TCGA-LIHC cohort with Spearman’s rank correlation test. As shown in **Figures 7A–C**, M1 macrophage marker PTGS2 showed the highest positive correlation with CXCL2 (r = 0.32, P < 0.001). In addition, we employed the GEPIA2 database to cross-validate the association and the results were similar to our previous finding (**Supplementary Figure S3**). This finding indicated that CXCL2 may regulate immune response by promoting the formation of the M1 macrophage.

## Discussion

This study aimed to explore critical and novel ferroptosis-related biomarkers for prognosis of HCC patients. Through three GEO datasets and a ferroptosis-related gene dataset, we screened six co-DEGs, including one up-regulated gene, ACSL4, and five down-regulated genes, STEAP3, MT1G, GCH1, HAMP, and CXCL2. We also found the low-expressed CXCL2 exhibited potential prognostic significance in patients with HCC, and low expression of CXCL2 was associated with malignancy clinical features, such as AFP > 400 ng/ml, nodal metastasis, and higher grades. Furthermore, *in vitro* experiments demonstrated that CXCL2 was down-regulated in HCC samples and the overexpression of CXCL2 inhibited cell proliferation. ssGSEA analysis revealed that enrichment of genes co-expressed with CXCL2 were mainly involved in inflammation and immune-related pathways. These findings provided a new perspective on CXCL2 as a prognostic marker in HCC.

Ferroptosis is characterized as a form of non-apoptotic regulated cell death driven by iron accumulation and lipid peroxidation (40, 41). Increasing evidence suggests that ferroptosis plays pivotal roles in tumor development and is strongly correlated with therapeutic responses in various cancer types (42, 43). Sorafenib, a multi-kinase inhibitor, remains the first-line targeted therapy for advanced HCC patients (44). Previously studies indicated that sorafenib exerted antitumor effects not only by inhibiting cell proliferation and inducing apoptosis, but also by antiangiogenic activity (45, 46). However, recent studies have shown that sorafenib may exert its antitumoral activity mainly by promoting ferroptosis by inhibiting the function of system Xc- (cystine/glutamate antiporter system) (47–49). High expression of ACSL4 (Acyl-CoA synthetase long chain family member 4), a driver of ferroptosis, was positively associated with the sensitivity of sorafenib in HCC (50). Combination of sorafenib and ferroptosis inducers may be a new and effective therapeutic strategy in HCC patients.

Chemokine CXCL2 is a small secreted protein with a Glu-Leu-Arg (ELR) motif that binds to CXC chemokine receptor 2 (CXCR2) to promote tumor angiogenesis and endothelial cell survival (51). According to a recent study by Linkermann et al. (52), the expression level of CXCL2 was significantly reduced upon the application of ferroptosis inhibitor ferrostatin-1 (Fer-1) in a mouse model of oxalate nephropathy. Ferroptosis inducer RLS3 increased the expression of CXCL2 in vascular smooth muscle cells (53). In this study, bioinformatics analysis and experimental validation confirmed the down-regulation of CXCL2 in HCC, and overexpression of CXCL2 increased intracellular ROS, Fe^2+^ and MDA levels. These results might provide further insights into the potential role of CXCL2 in mediating ferroptosis. The ROC curve, based on a series of cut-off points with sensitivity and specificity, is an effective method to evaluate the performance of diagnostic tests (54, 55). In our study, we found the area under the ROC curve (AUC) for CXCL2 to diagnose HCC reached 0.903, indicating a promising clinical diagnostic significance of CXCL2 which needed further clinical validation.

An accumulating body of evidence suggests that immune microenvironment affects tumor development and response to therapy (56–58). Single-cell RNA sequencing analysis revealed the immunosuppressive landscape in HCC patients (59). Checkpoint blockade immunotherapies have redefined cancer treatment paradigm (60). The combination therapy of atezolizumab (anti-PD-L1) and bevacizumab (anti-VEGF) improved overall survival in patients with HCC compared to sorafenib, leading to FDA approval of this regimen (3, 61). Consistent with previous findings, ferroptosis-related gene CXCL2 was down-regulated in HCC samples compared with adjacent normal tissues and overexpression of CXCL2 could inhibit cell proliferation (19, 62). However, previous studies mainly focused on apoptosis pathways. In this paper, ssGSEA showed that co-expression genes of CXCL2 were mainly enriched in inflammation and immune-associated pathways, such as acute inflammatory response, humoral immune response, adaptive immune response. The interaction analysis between CXCL2 and immune system further indicated that CXCL2 expression was positively correlated with lymphocytes, including neutrophils and macrophages, especially the M1 macrophages (anti-tumor). The positive correlation was also found between CXCL2 and immunostimulators, such as IL-16, CD40LG, CD48, and TNFSF14. In addition, correlation analysis between CXCL2 and immune infiltration score in GSE14520 dataset indicated that patients with high immune infiltration score had higher CXCL2 expression and better prognosis. Together, these findings suggested that ferroptosis-related gene CXCL2 may regulate tumor immune response to influence cancer development and serve as a biomarker for diagnosis and prognosis in patients with HCC.

## Conclusion

Conclusively, our study provides a novel insight into the biological role of CXCL2 and its interaction with immune microenvironment in HCC patients. CXCL2 was down-regulated in HCC tissues and cell lines, and overexpression of CXCL2 could inhibit cell proliferation. High expression of CXCL2 exhibited a favorable prognostic indicator in patients with HCC. Furthermore, CXCL2 expression was obviously correlated with the immune signatures, including tumor-infiltrating immune cells and immunostimulators. Therefore, our findings suggest ferroptosis-related gene CXCL2 plays a pivotal role in the development of HCC by regulating immune response and may be a promising diagnostic and prognostic indicator in patients with HCC.

## Data availability statement

The datasets presented in this study can be found in online repositories. The names of the repository/repositories and accession number(s) can be found in the article/**Supplementary Material**.

## Author contributions

QY and YY: Acquisition of data. QY and YY: Analysis and interpretation of data. YY: Conception and design. QL: Data curation. QL and YL: Development of methodology. QY and ZG: Writing and/or revising the manuscript. All authors contributed to the article and approved the submitted version.

## Funding

This study is supported by grants from the Science and Technology Innovation Program of Hunan Province (2021RC3029), the horizontal project (2022, 143010100; 2021-021, 143010100), the China Postdoctoral Science Foundation (2021T140754, 2020M672521) and the Postdoctoral Science Foundation of Central South University (248485).

## Conflict of interest

The authors declare that the research was conducted in the absence of any commercial or financial relationships that could be construed as a potential conflict of interest.

## Publisher’s note

All claims expressed in this article are solely those of the authors and do not necessarily represent those of their affiliated organizations, or those of the publisher, the editors and the reviewers. Any product that may be evaluated in this article, or claim that may be made by its manufacturer, is not guaranteed or endorsed by the publisher.

## Supplementary material

Supplementary Table 1 Differentially expressed genes in GSE6764, GSE14323, and GSE14520.

Supplementary Figure 1 Prognostic values of CXCL2, GCH1, and HAMP in HCC with the GEPIA2 database. **(A-F)** Kaplan-Meier curves for overall survival and disease-free survival of CXCL2, GCH1, and HAMP in patients with HCC by the GEPIA2 database.

Supplementary Figure 2 Functional enrichment analysis of CXCL2-associated network. **(A, B)** GO and KEGG enrichment analysis of CXCL2-associated network in TCGA-LIHC cohort with the Xiantao database.

Supplementary Figure 3 The association between CXCL2 expression and classical macrophage phenotype markers in TCGA-LIHC cohort. **(A)** Scatter plots showing the association between CXCL2 and M0 (undifferentiated) macrophage markers (AIF1). **(B-E)** Scatter plots showing the association between CXCL2 and M1 (anti-tumor) macrophage markers (IL12A, TNF, NOS2, PTGS2). **(F-I)** Scatter plots showing the association between CXCL2 and M2 (tumor-promoting) macrophage markers (IL10, CD163, TGFB1, CSF1R).
