# Supplementary material for: Application of genomic selection and experimental techniques to predict cell death and immunotherapeutic efficacy of ferroptosis-related CXCL2 in hepatocellular carcinoma
Source: Front Oncol. 2022 Oct 5;12:998736. doi: 10.3389/fonc.2022.998736 (PMC9579367; doi:10.3389/fonc.2022.998736)
Supplement: Supplementary file 1 [file Table_2.docx]

| Characteristics | Total (N) | Odds Ratio (OR) | P value |
| --- | --- | --- | --- |
| T stage (T3&T4 vs. T1&T2) | 371 | 0.723 (0.449-1.158) | 0.179 |
| N stage (N1 vs. N0) | 258 | 0.390 (0.019-3.094) | 0.418 |
| M stage (M1 vs. M0) | 272 | 0.405 (0.020-3.208) | 0.436 |
| Histologic grade (G3&G4 vs. G1&G2) | 369 | 0.651 (0.424-0.995) | 0.048 |
| Tumor status (With tumor vs. Tumor free) | 355 | 1.156 (0.759-1.761) | 0.500 |
| Gender (Male vs. Female) | 374 | 1.309 (0.848-2.026) | 0.225 |
| Age (>60 vs. <=60) | 373 | 1.700 (1.130-2.567) | 0.011 |
| Race (White vs. Asian&Black or African American) | 362 | 1.998 (1.318-3.045) | 0.001 |
| Pathologic stage (Stage III&Stage IV vs. Stage I&Stage II) | 350 | 0.729 (0.449-1.179) | 0.199 |
| Residual tumor (R1&R2 vs. R0) | 345 | 1.681 (0.646-4.668) | 0.295 |
| Adjacent hepatic tissue inflammation (Mild&Severe vs. None) | 237 | 1.017 (0.611-1.693) | 0.949 |
| AFP (ng/ml) (>400 vs. <=400) | 280 | 0.265 (0.142-0.480) | <0.001 |
| Albumin (g/dl) (<3.5 vs. >=3.5) | 300 | 1.711 (0.994-2.985) | 0.055 |
| Vascular invasion (Yes vs. No) | 318 | 0.785 (0.493-1.247) | 0.306 |
| Fibrosis ishak score (3/4&5/6 vs. 0&1/2) | 215 | 1.096 (0.642-1.874) | 0.737 |
| Child-Pugh grade (B&C vs. A) | 241 | 0.577 (0.222-1.402) | 0.235 |
| Prothrombin time (>4 vs. <=4) | 297 | 1.478 (0.898-2.448) | 0.126 |

Supplementary Table S2. Demographic characteristics of CXCL2 expression in HCC.
