# Supplementary material for: Application of genomic selection and experimental techniques to predict cell death and immunotherapeutic efficacy of ferroptosis-related CXCL2 in hepatocellular carcinoma
Source: Front Oncol. 2022 Oct 5;12:998736. doi: 10.3389/fonc.2022.998736 (PMC9579367; doi:10.3389/fonc.2022.998736)
Supplement: Supplementary file 2 [file Table_3.docx]

Supplementary Table S3. The top 50 genes positively associated with CXCL2 in LinkedOmics

| CXCL2 | NNMT | FGA | CEBPD | FGG | FGB | GADD45B | SOD2 | SAA2 | SERPINA3 |
| --- | --- | --- | --- | --- | --- | --- | --- | --- | --- |
| C9 | FAM83A-AS1 | SAA1 | C1R | ORM1 | NAMPT | HP | DHODH | IFITM2 | LRG1 |
| CSRNP1 | ETS2 | PLGLB2 | LBP | CFI | STOM | CRP | GCH1 | CEBPB | MTHFD2L |
| C1S | ITIH4 | CXCL1 | JUNB | ZC3H12A | C19orf66 | C3P1 | SDS | SOCS3 | C8B |
| IL32 | SERPING1 | AGXT2 | BCL3 | C5AR1 | CP | CCDC71L | DUSP1 | DNAH5 | CFB |
