# Supplementary material for: Application of genomic selection and experimental techniques to predict cell death and immunotherapeutic efficacy of ferroptosis-related CXCL2 in hepatocellular carcinoma
Source: Front Oncol. 2022 Oct 5;12:998736. doi: 10.3389/fonc.2022.998736 (PMC9579367; doi:10.3389/fonc.2022.998736)
Supplement: Supplementary file 3 [file Table_4.docx]

Supplementary Table S4. The top 50 genes negatively associated with CXCL2 in LinkedOmics

| CEP250 | ZNF585A | PIP4K2B | CCDC97 | SKP2 | LSM14B | CSE1L | IPO9 | ZBTB12 | CHKA |
| --- | --- | --- | --- | --- | --- | --- | --- | --- | --- |
| ZNF765 | TRIM24 | SMARCC1 | ZNF28 | KHSRP | PPP2R5D | NCOA6 | ZNF845 | DVL3 | SOX12 |
| LIN9 | ZNF761 | PSPH | CBFA2T2 | ZNF687 | AGBL5 | ZNF473 | ZNF606 | TM9SF4 | TMEM169 |
| DPF2 | ZBED8 | ZNF256 | XRCC1 | NELFE | PRR12 | ATXN7L3B | TCF3 | SAP130 | ZNF836 |
| SGOL2 | GPATCH1 | SMPD4 | RFC3 | ZNF552 | RPS6KC1 | ZNF431 | MSH2 | CKAP5 | NRBP1 |
