# Supplementary figures and images for: Application of genomic selection and experimental techniques to predict cell death and immunotherapeutic efficacy of ferroptosis-related CXCL2 in hepatocellular carcinoma
Source: Front Oncol. 2022 Oct 5;12:998736. doi: 10.3389/fonc.2022.998736 (PMC9579367; doi:10.3389/fonc.2022.998736)

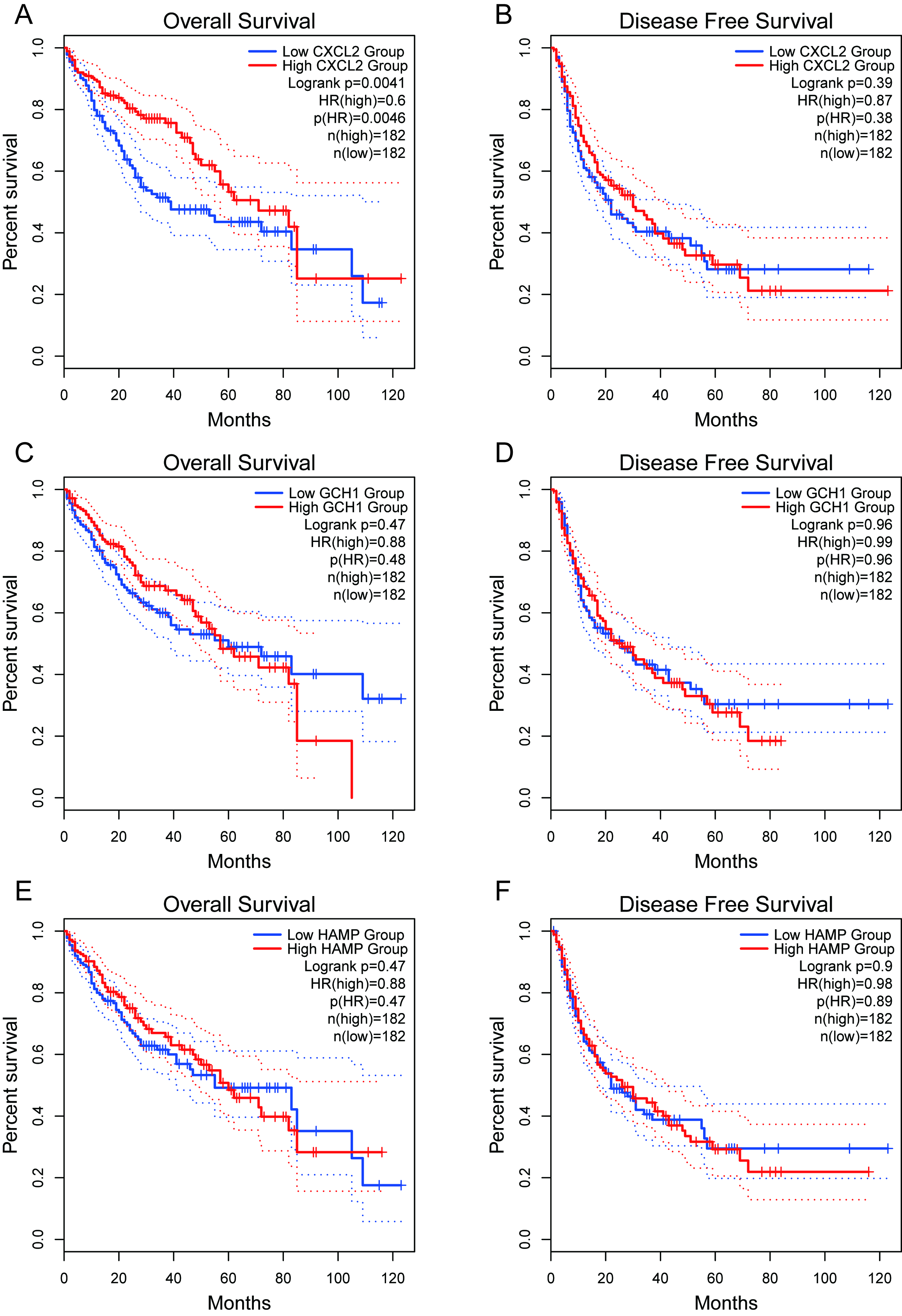

Supplement: Supplementary Figure 1 — Prognostic values of CXCL2, GCH1, and HAMP in HCC with the GEPIA2 database. (A-F) Kaplan-Meier curves for overall survival and disease-free survival of CXCL2, GCH1, and HAMP in patients with HCC by the GEPIA2 database. [file Image_1.tif]

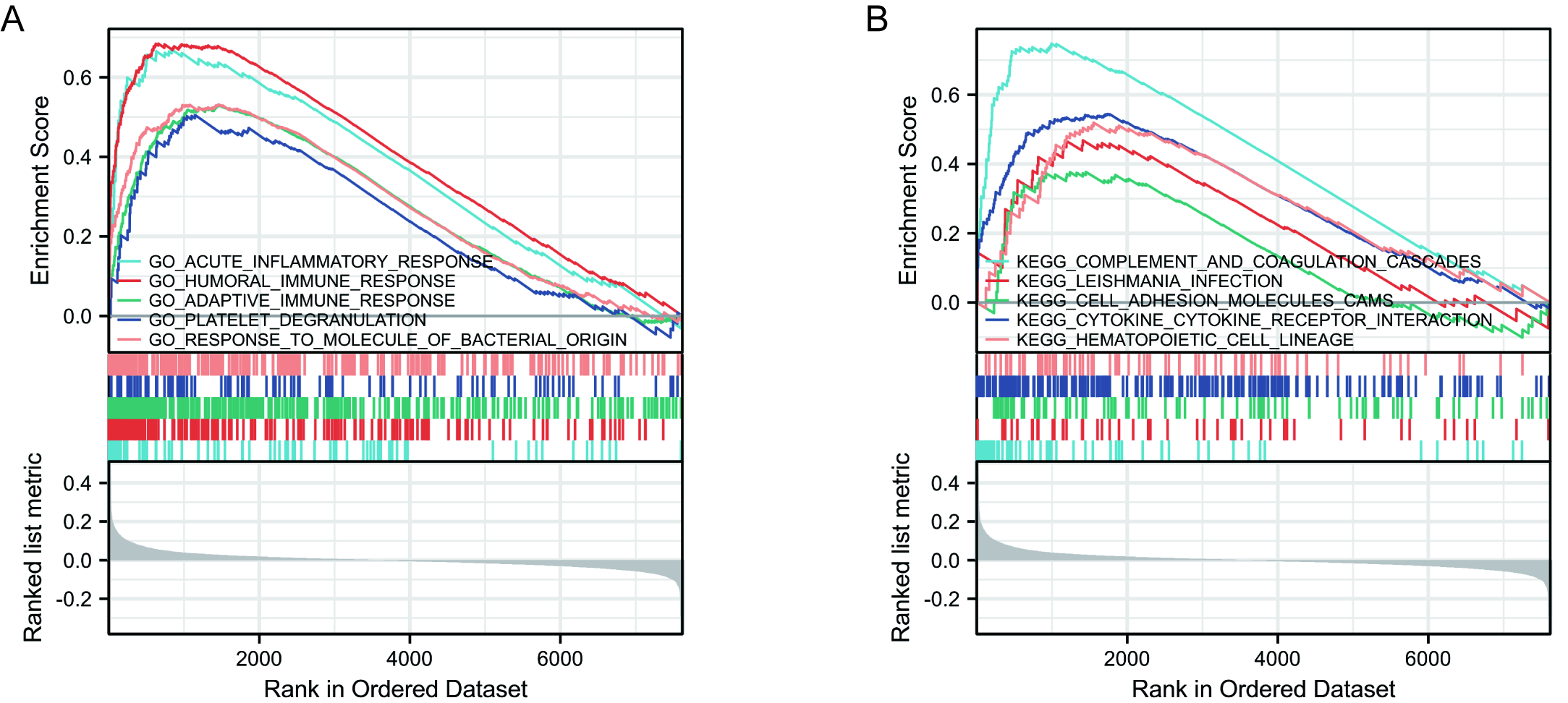

Supplement: Supplementary Figure 2 — Functional enrichment analysis of CXCL2-associated network. (A, B) GO and KEGG enrichment analysis of CXCL2-associated network in TCGA-LIHC cohort with the Xiantao database. [file Image_2.tif]

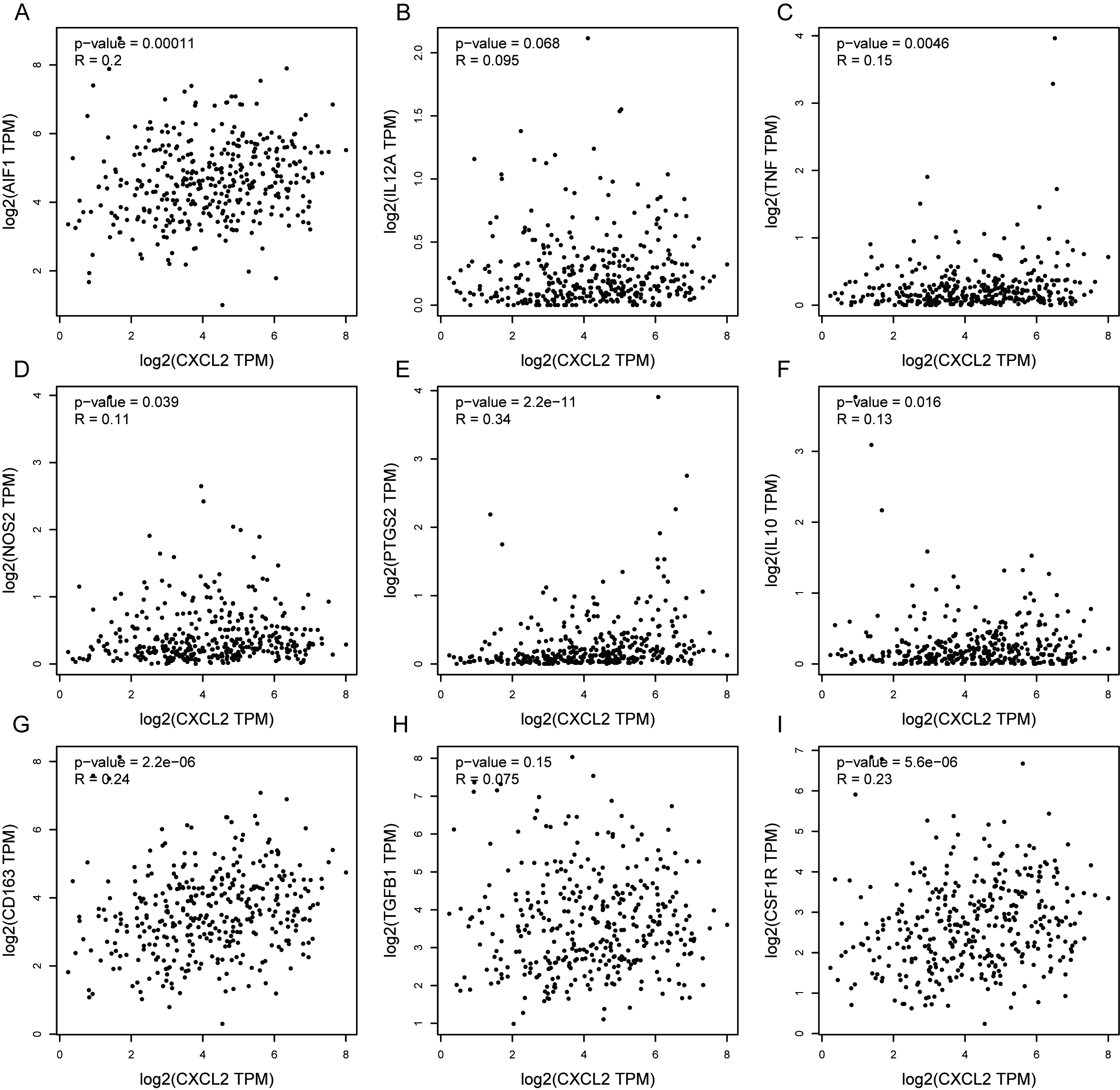

Supplement: Supplementary Figure 3 — The association between CXCL2 expression and classical macrophage phenotype markers in TCGA-LIHC cohort. (A) Scatter plots showing the association between CXCL2 and M0 (undifferentiated) macrophage markers (AIF1). (B-E) Scatter plots showing the association between CXCL2 and M1 (anti-tumor) macrophage markers (IL12A, TNF, NOS2, PTGS2). (F-I) Scatter plots showing the association between CXCL2 and M2 (tumor-promoting) macrophage markers (IL10, CD163, TGFB1, CSF1R). [file Image_3.tif]
